# A Phase 1 Study of IRX195183, a RARα-Selective CYP26 Resistant Retinoid, in Patients With Relapsed or Refractory AML

**DOI:** 10.3389/fonc.2020.587062

**Published:** 2020-10-23

**Authors:** Alexander J. Ambinder, Kelly Norsworthy, Daniela Hernandez, Laura Palau, Bogdan Paun, Amy Duffield, Rosh Chandraratna, Martin Sanders, Ravi Varadhan, Richard J. Jones, B. Douglas Smith, Gabriel Ghiaur

**Affiliations:** ^1^Department of Oncology, Sidney Kimmel Comprehensive Cancer Center, Johns Hopkins University School of Medicine, Baltimore, MD, United States; ^2^Department of Pathology, Johns Hopkins University School of Medicine, Baltimore, MD, United States; ^3^IO Therapeutics, Santa Ana, CA, United States; ^4^Division of Biostatistics and Bioinformatics, Johns Hopkins/Sidney Kimmel Comprehensive Cancer Center, Baltimore, MD, United States

**Keywords:** acute myeloid leukemia, retinoic acid receptor agonist, differentiation therapy, microenvironment niche, phase 1 clinical trial

## Abstract

Subsets of non-acute promyelocytic leukemia (APL) acute myelogenous leukemia (AML) exhibit aberrant retinoid signaling and demonstrate sensitivity to retinoids *in vitro*. We present the results of a phase 1 dose-escalation study that evaluated the safety, pharmacodynamics, and efficacy of IRX195183, a novel retinoic acid receptor α agonist, in patients with relapsed or refractory myelodysplastic syndrome (MDS) or AML. In this single center, single arm study, eleven patients with relapsed or refractory MDS/AML were enrolled and treated. Oral IRX195183 was administered at two dose levels: 50 mg daily or 75 mg daily for a total of two 28-day cycles. Patients with stable disease or better were allowed to continue on the drug for four additional 28-day cycles. Common adverse events included hypertriglyceridemia, fatigue, dyspnea, and edema. Three patients at the first dose level developed asymptomatic Grade 3 hypertriglyceridemia. The maximally tolerated dose was not reached. Four of the eleven patients had (36%) stable disease or better. One had a morphological complete remission with incomplete hematologic recovery while on the study drug. Two patients had evidence of *in vivo* leukemic blast maturation, as reflected by increased CD38 expression. In a pharmacodynamics study, plasma samples from four patients treated at the lowest dose level demonstrated the capacity to differentiate leukemic cells from the NB4 cell line *in vitro*. These results suggest that IRX195183 is safe, achieves biologically meaningful plasma concentrations and may be efficacious in a subset of patients with MDS/AML. **Clinical Trial Registration:** clinicaltrials.gov, identifier NCT02749708.

## Introduction

Acute myeloid leukemia (AML) is a clonal process that arises out of sequential genetic and epigenetic alterations that cause an arrest of differentiation and unfettered proliferation. The retinoid signaling pathway is essential to hematopoietic differentiation and its disruption can contribute to leukemogenesis (1). This is exemplified by the t(15;17) translocation in acute promyelocytic leukemia (APL) that juxtaposes the promyelocytic leukemia protein (*PML*) gene with the retinoic acid receptor α gene. The resulting PML-RARA fusion protein acts as a dominant negative, disrupting the activities of WT RARα and RARγ, leading to a block in differentiation at the level of the promyelocyte (2). All-trans-retinoic acid (ATRA) is a natural retinoid that restores normal retinoic acid signaling and promotes the differentiation of leukemic blasts. It now forms the cornerstone of APL treatment.

Aberrant retinoic acid signaling has been demonstrated in other subsets of AML (3–5). *In vitro*, ATRA is capable of inducing differentiation and apoptosis of most non-APL AML cell lines (6). Furthermore, expression profiling predicts *in vitro* sensitivity to retinoid analogs (4). In clinical trials, however, ATRA has not shown consistent clinical benefit in non-APL AML (7–13).

These lackluster results may be a result of the unique pharmacologic properties of ATRA. ATRA induces its own catabolism by stimulating upregulation of its metabolizing enzyme, CYP26, which is most prominently expressed in the liver (14–17); this leads to a reduction in the C_max_ of ATRA with prolonged administration. CYP26 is also produced by the bone marrow stroma where it plays an essential role in endogenous retinoid homeostasis (6, 17, 18). Stromal CYP26 establishes a low retinoid environment within the stem cell niche, which is necessary for the maintenance of a pool of quiescent, long term hematopoietic stem cells (HSCs) (18). In the setting of AML, stromal upregulation of CYP26 in response to exogenous ATRA shields leukemic stem cells (LSCs) from its differentiating effects (17).

IRX195183 is a synthetic retinoid that specifically binds to the retinoic acid receptor α and is resistant to metabolism by CYP26 (17). Pharmacokinetics from the first in-human trial of IRX195183 demonstrated a steady improvement of C_max_ and AUC from D0 to D28 across a range of doses from 15mg/m^2^/day to 60mg/m^2^/day (described in the publicly available study protocol)^1^. This contrasts with the blunted rise in C_max_ and AUC that is observed with prolonged ATRA administration (19). suggesting that IRX195183 does not induce its own auto-catabolism. In both *in vitro* and *in vivo* mouse models, the effects of ATRA are attenuated by the presence of CYP26-producing mesenchymal stromal cells, whereas the effects of IRX195183 are unmitigated (17).

IRX195183’s superior pharmacokinetic and pharmacodynamic profile make it a promising drug for the treatment of patients with myeloid malignancies. The previously conducted phase 1 study of the drug was performed in patients with hepatocellular carcinoma. Grade 3 elevations of transaminases were frequently observed in that study, but accurate attribution of this adverse effect was complicated by the high burden of liver disease in this patient population. We designed a phase I/II study of IRX195183 in the treatment of relapsed and refractory high-grade myelodysplastic syndrome (MDS) and AML. Due to the unavailability of the drug, we were unable to proceed with the phase 2 expansion cohort. Herein, we describe the design and results of the phase I portion of the study.

## Materials and Methods

The clinical trial registration number is NCT02749708. The study protocol was approved by the Johns Hopkins Institutional Review Board and was conducted in accordance with the Declaration of Helsinki.

### Patient Population

Patients between the ages of 18 and 60 years of age with pathologically confirmed AML, MDS, chronic myelomonocytic leukemia (CMML), or MDS/MPN overlap syndrome were considered for enrollment. AML patients must have had either 1) relapsed or refractory disease after receiving one or more courses of induction chemotherapy, hypomethylating agent therapy, or bone marrow transplant or 2) *de novo* AML not deemed to be a candidate for conventional therapy based on age, co-morbidities, or patient preference. Patients with MDS, CMML, or MDS/MPN had to have high-risk features, defined as follows: 1) relapsed after initial response or were refractory after receiving at least four cycles of HMA therapy or 2) had *de novo* MDS but refused HMA therapy. High-risk features included at least one of the following: International Prognostic Scoring System (IPSS) of INT-2 or higher; Revised-IPSS (IPSS-R) of high or very high; secondary MDS; INT-1 IPSS or INT R-IPSS with excess blasts (≥5% blasts on bone marrow biopsy) or with transfusion dependency; or CMML or MDS/MPN with excess blasts, transfusion-dependency, abnormal karyotype, or proliferative features.

Patients were required to have an ECOG ≤ 2 or Karnofsky ≥ 60%; creatinine level of 3mg/dL or lower; total bilirubin ≤ 3mg/dL unless due to Gilbert’s syndrome, hemolysis or ineffective hematopoiesis; AST (SGOT) and ALT (SGPT) ≤ 3 x the upper limit of normal (ULN); white blood cell (WBC) count ≤10,000/μl; and if the patient was a woman of childbearing age, they were required to have a negative serum or urine pregnancy test within 72 h of the start of the study drug. Patients who had received prior therapies were required to undergo a 3-week washout period and had to have recovered from all toxicities prior to the initiation of therapy. Patients requiring hydroxyurea to bring the WBC below 10,000/μl were required to have a 48 h wash out period.

Major exclusion criteria included any serious medical condition or uncontrolled concurrent illness including active infection, symptomatic congestive heart failure, unstable angina pectoris, cardiac arrhythmia, or laboratory abnormality that would limit compliance with the study requirements or preclude informed consent from being obtained; leukemic involvement of the central nervous system (CNS); pulmonary leukostasis; or disseminated intravascular coagulation (DIC).

### Study Design and Treatment

**Figure 1:**
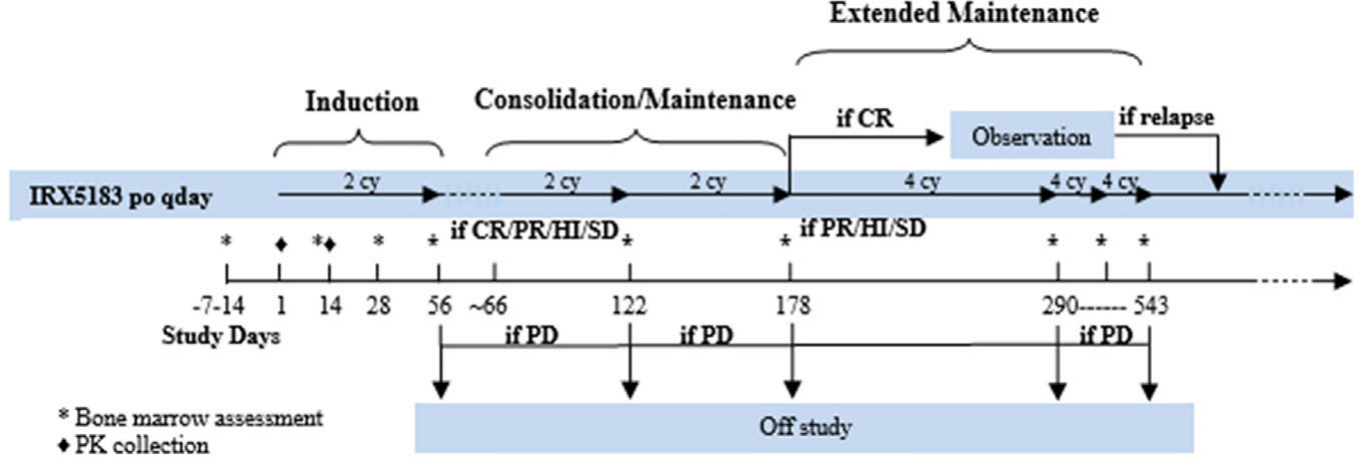
Treatment schema of phase I clinical trial using IRX195183 in patients with high risk MDS and relapsed/refractory AML.

This was a single-center, single-arm prospective phase I study. The treatment schema is summarized in **Figure 1**. The dose escalation phase used a 3 + 3 design with three dose levels (DL): DL1 was 50mg, DL2 was 75 mg, and DL3 was 100 mg. For induction therapy, IRX195183 was administered orally once daily for two 28-day cycles. Patients who had stable disease (SD) or better were eligible to continue on consolidation/maintenance, which consisted of up to four continuous 28-day cycles using the same dose they had previously received. There was no intra-patient dose escalation. IRX5183 was provided by Io Therapeutics, Inc.

### Assessment of Safety and Efficacy

The primary objective was to evaluate the safety and toxicity of IRX195183 and to determine the recommended phase 2 dose. The primary endpoint was toxicity of IRX195183, determined by grading a tabulation using the National Cancer Institute Common Toxicity Criteria, Version 5.0, in order to determine dose-limiting toxicities (DLTs) and the recommended phase 2 dose. DLTs were defined as Grade 3 or higher non-hematologic toxicities, any toxicity that resulted in a delay of therapy for 2 weeks or greater, and Grade 4 treatment-associated aplasia that persists beyond 4 weeks. Exceptions included transient laboratory abnormalities that could be treated or resolved to Grade 2 or less within 1 week from holding the study drug, toxicities associated with differentiation syndrome that resolved within 2 weeks of steroid therapy to Grade 1, and asymptomatic Grade 3 hypertriglyceridemia. Grade 3 hypertriglyceridemia was initially considered a DLT, but the protocol was later amended to exclude asymptomatic Grade 3 hypertriglyceridemia. Patients with Grade 3 hypertriglyceridemia were allowed to continue on study after initiating treatment with the triglyceride lowering agent gemfibrozil.

Efficacy was a secondary endpoint of phase I and patients who received at least 1 dose were included in efficacy analyses. Bone marrow response assessments were performed at baseline and then after each cycle. Clinical responses were defined according to the Modified International Working Group-2003 criteria for AML (20) and the Modified International Working Group-2006 criteria for MDS (21).

### Processing of Patient Blood Samples

Whole blood samples were centrifuged at 500 g in a clinical centrifuge, and the plasma layer was carefully removed, aliquoted, and stored at -80°C. Frozen plasma samples were thawed and clarified by centrifugation at 16,000 *g* for 2 min.

### Cell Culture

The mouse mesenchymal stroma OP9 cells were cultured in in α-MEM with 2 mM L-glutamine (Life Technologies, Thermo Fisher Scientific), 100 μg/ml penicillin-streptomycin (P/S) (Gibco, Thermo Fisher Scientific), and 20% fetal calf serum (FCS) (Sigma-Aldrich). The APL cells from the NB4 cell line were cultured in RPMI 1640, L-glutamine, P/S, and with 10% and 20% FCS (R20). The cell line was purchased from ATCC and recently tested negative for Mycoplasma.

### Co-Culture System

The co-culture model has previously been described (6, 22). Briefly, 24-well plates were coated with 0.1% gelatin (Sigma-Aldrich) in PBS for at least 30 min at 37˚C. The gelatin was then removed and 5x10^4^ stromal cells per well were cultured overnight to obtain a confluent monolayer. At that time, media was removed, and leukemia cells were added to the culture at a density of 5x10^4^ cells/well and incubated with in various conditions for 72 h.

### Flow Cytometry for Bone Marrow Aspirate Samples

Flow cytometric immunophenotyping was performed on fresh bone marrow aspirates. The material was collected in EDTA or heparin anticoagulant and processed routinely using an RBC lysis method. Cell suspensions were incubated with combinations of monoclonal antibodies (Becton Dickinson) that were used at concentrations titrated for optimal staining. Specimens were subjected to a myeloid leukemia panel that included CD7, CD10, CD11b, CD13, CD14, CD15, CD16, CD33, CD34, CD38, CD45, CD56, CD64, CD71, CD117, CD123, and HLA-DR. CD38 was conjugated to PerCP-Cy5.5. The data were analyzed on BD FACSCanto 10-color system (BD Biosciences). List mode data files were acquired and analyzed for each specimen using BD FACSDiva (BD Biosciences) and Infinicyt (Cytognos) for data acquisition and analysis, respectively.

### Flow Cytometry for Cell Lines

Cell lines in the plasma differentiation assay were analyzed for expression of cell surface antigens using FACS Calibur (BD Biosciences, CA, USA). Treated cells were removed from the plate, washed with PBS and counted using Trypan Blue. For flow cytometry, cells were washed with PBS containing 0.2% BSA (Sigma-Aldrich) and stained with the appropriate antibodies for 15 min at room temperature (PE-conjugated mouse anti-human CD11b IgG1, FITC-conjugated mouse anti-human CD34 IgG1, PE-conjugated mouse anti-human CD38 IgG2α, and APC-conjugated mouse anti-human CD45 IgG2β antibodies, BD Biosciences). All antibodies were purchased from BD Biosciences. Cells were then washed with PBS and evaluated by FACS Calibur (BD Biosciences) with a minimum acquisition of 10,000 events.

### *In Vivo* Induction of Terminal Differentiation

We performed flow cytometry to assess CD11b and CD38 expression, markers of terminal differentiation, on bone marrow biopsy specimens obtained at baseline and after each cycle of therapy for every patient enrolled in the study.

### Plasma Differentiation Assay

Blood samples were collected from the first four patients enrolled in the study on day 14 of treatment, 2 h after the dose of IRX195183 was administered. NB4 cells were cultured in RPMI 1640 supplemented with L-glutamine, P/S and with 10% (R10) complete media supplemented with either 1%, 5%, or 10% patient plasma in the presence or absence of OP9 stroma cells. The degree of differentiation was then determined by measuring CD11b expression by flow cytometry and clonogenic activity in a colony forming assay. Data were normalized to NB4 cells incubated in plasma from untreated controls. For comparison, we used plasma derived from three patients with APL treated with ATRA per standard of care.

### Statistical Analysis

Pre-clinical data were analyzed using unpaired, 2-tailed Student’s t tests to compare differences between drug treatment groups.

## Results

### Patient Characteristics

**Figure 2:**
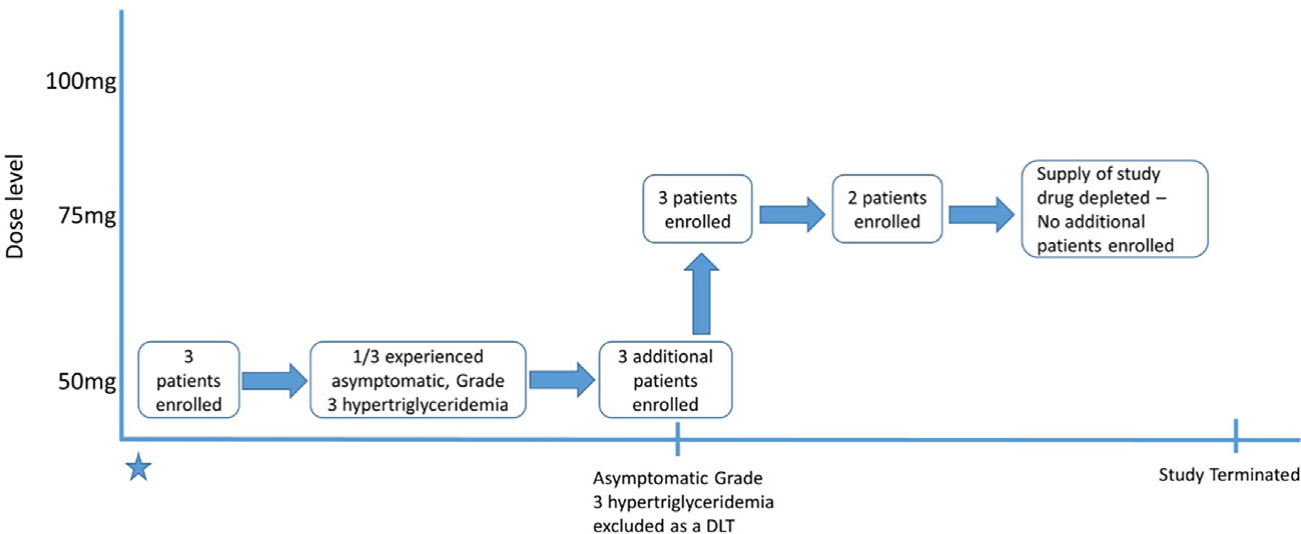
Flow chart diagramming patient enrollment. DLT, Dose-limiting toxicity.

Eleven patients were enrolled between February 2017 and August 2018. The initial cohort of three patients received IRX195183 50 mg daily in 28-day cycles. A flow chart for the study conduct is displayed in **Figure 2**. One patient had asymptomatic Grade 3 hypertriglyceridemia triggering the enrollment of three more patients at the same dose level. The protocol was then amended to exclude asymptomatic Grade 3 hypertriglyceridemia as a DLT. Symptomatic Grade 3 hypertriglyceridemia and any Grade 4 hypertriglyceridemia remained DLTs. Using this new definition of DLT, patients were then enrolled at the next dose level.

Patient characteristics are summarized in **Table 1**. The average age was 69 years and 55% of the patients were male. Eight patients had secondary AML. The remaining three patients had primary AML, MDS with excess blasts, and CMML. All but one patient had received at least one line of prior standard-of-care therapy.

**Table 1:** Baseline patient characteristics.

Patient	Dose level	Age	Gender	Diagnosis	Disease Status	No. of Prior Therapies	ECOG	Karyotype	Molecular Mutations	Days on trial	Response Duration	In Vivo Maturation	Best Response	Reason for Discontinuation
1	50 mg	70	Male	sAML	Refractory	1	1	Monosomy 7	SETBP1, ASXL1, U2AF1	48	19	No	SD	Infection
2	50 mg	58	Female	t-MN	Relapsed	2	1	11q23	FLT3, ASXL1, NUP98	56	N/A	No	PD	Progressive disease
3	50 mg	67	Female	sAML	Relapsed	4	1	5q-, trisomy 8	RUNX1, TET2, BCOR, DNMT3A, U2AF1	34	N/A	Yes	PD	Progressive disease
4	50 mg	58	Male	sAML	Relapsed	3	0	Normal	RUNX1, IDH2, DNMT3A	64	N/A	No	PD	Progressive disease
5	50 mg	75	Male	sAML	Refractory	1	1	Complex	SRSF2, TET2	69	42	Yes	CRi	Hypertriglyceridemia
6	50 mg	76	Male	CMML-2	Relapsed	2	1	Normal	ASXL1, SRSF2, TET2	63	48	No	SD	Hypertriglyceridemia
7	75 mg	64	Female	sAML	Relapsed	4	1	Normal	NPM1	63	N/A	No	PD	Progressive disease
8	75 mg	70	Male	MDS-EB1	Refractory	1	1	11q23	ETV6, ASXL1, NF1, SRSF2, KIT	112	85	No	SD	Progressive disease
9	75 mg	78	Female	sAML	Untreated	0	1	Normal	SF3B1, BCOR, BCORL1, PTPN11, RUNX1	16	N/A	No	N/A*	Infection
10	75 mg	77	Male	sAML	Relapsed	2	1	5q-	Not tested	4	N/A	No	N/A*	Infection
11	75 mg	68	Female	AML	Relapsed	2	1	5q-	p53	43	N/A	No	PD	Progressive disease

*not applicable due to inadequate duration of therapy to assess response; SD, Stable disease; PD, Progressive disease; LR, Lineage response.

Patients were assessed regularly for safety as well as disease status. All six patients in DL1 completed at least one cycle of therapy and could be assessed for tolerability and response. After the DL1 cohort was completed and the drug was deemed to be safe at this level, three patients were enrolled in the DL2 cohort and received IRX195183 75 mg daily. One of the three went off study due to infection before DLTs could adequately be assessed, so two additional patients were enrolled before the study was terminated due to inadequate supply of the study drug. No dose escalations or reductions were made during the course of the trial for any individual patient.

### Safety and Toxicity

**Table 2** summarizes the treatment-emergent adverse events at least possibly related to IRX195183. A total of six patients developed hypertriglyceridemia. Three patients had Grades 1–2 hypertriglyceridemia and three had Grades 3–4 hypertriglyceridemia. Five of the six patients were treated with lipid modifying therapies including gemfibrozil and omega-3-acid ethyl esters. Once initiated, lipid modifying therapies were continued until the study drug was discontinued. For all three patients with Grade 3 hypertriglyceridemia, the study drug was held until the hypertriglyceridemia improved. One patient was able to resume therapy after initiation of after initiating lipid modifying therapy, whereas hypertriglyceridemia led to permanent discontinuation of the study drug in the remaining two patients. All three patients who experienced grade 3 hypertriglyceridemia were treated at the first dose level, whereas none of the patients treated at the second dose level developed Grades 3–4 hypertriglyceridemia. All six patients with hypertriglyceridemia were asymptomatic and none suffered adverse consequences such as hepatosplenomegaly, acute pancreatitis, dyspnea, or neurological symptoms. There were no other DLTs and no other patients discontinued the study due to study drug-related adverse events.

**Table 2:** Treatment emergent adverse events possibly related to IRX195183.

	N = 11	
Adverse Effect:	Grade 1/2, n (%)	Grade 3/4, n (%)
Asymptomatic Hypertriglyceridemia	3 (27%)	3 (27%)
Fatigue	2 (18%)	3 (27%)
Dyspnea	3 (27%)	1 (9%)
Edema	3 (27%)	0
Hypoxia	1 (9%)	1 (9%)
Pneumonia	1 (9%)	1 (9%)
Elevated Transaminases	1 (9%)	1 (9%)
Hyponatremia	1 (9%)	1 (9%)
Anorexia	2 (18%)	0
Arthralgias	2 (18%)	0
Hypocalcemia	2 (18%)	0
Nausea/Vomiting	2 (18%)	0
Cough	1 (9%)	0
Diarrhea	1 (9%)	0
Differentiation Syndrome	1 (9%)	0
Flushing	1 (9%)	0
Headache	1 (9%)	0
Hyperglycemia	1 (9%)	0
Hypertension	1 (9%)	0
Hyperuricemia	1 (9%)	0
Hypoalbuminemia	1 (9%)	0
Hypophosphatemia	1 (9%)	0
Increased cholesterol	1 (9%)	0
Increased creatinine	1 (9%)	0
Insomnia	1 (9%)	0
Lymphedema	1 (9%)	0
Pruritus	1 (9%)	0
Rash	1 (9%)	0
Tachycardia	1 (9%)	0

Patient 2 developed Grade 3 elevations of her transaminases in the setting of hospitalization, but they returned to normal levels without discontinuation of the study drug. Two patients developed leukocytosis (>10,000/cc mm) for which hydroxyurea was initiated. Another patient, Patient 2, developed Grade 3 hypoxia for which differentiation syndrome (DS) was considered a possible etiology. Her shortness of breath was accompanied by a small pleural effusion, but no other manifestations of DS. She was briefly treated with steroids and furosemide while the study drug was continued, and she clinically improved. Patient 6 developed grade 3 hyponatremia with a nadir sodium of 129 mmol/L. The patient’s hyponatremia was preceded by several days of poor oral intake and it resolved with administration of IV fluids. Two patients experienced Grade 3 fatigue. The remainder of the Grade 3–4 AEs were hematologic and attributed to the underlying leukemia. There were no serious adverse events. Five patients died within 30 days of withdrawal from the study. Two deaths occurred as a result of infection and three were attributed to progressive leukemia.

### Responses

**Figure 3:**
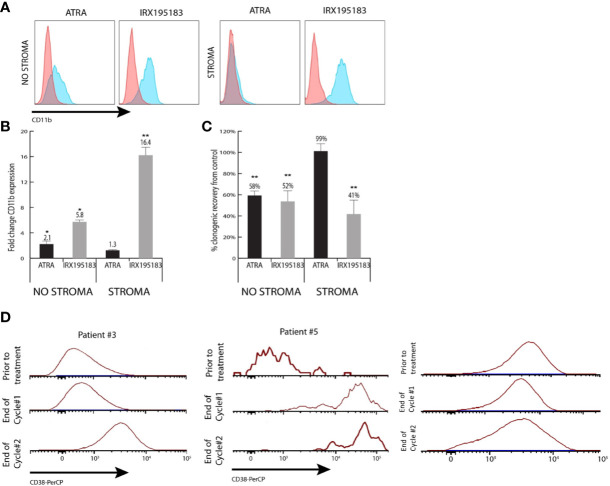
Differentiation activity of plasma from patients treated with IRX195183. **(A)** Representative flow plots of CD11b expression of NB4 cells cultured in the presence of 10% plasma from patients treated with ATRA (1^st^ and 3^rd^) or IRX195183 (2^nd^ and 4^th^) in the presence or absence of bone marrow stroma. **(B, C)** Differentiation activity on plasma from patients treated with either ATRA or IRX195183 as measured by upregulation of CD11b expression **(B)** and decreased clonogenic activity **(C)** of NB4 cells. Data represents mean ± SEM, n = 4 independent patients, *p < 0.05, **p < 0.01. P was calculated using unpaired, 2-tailed Student’s t test. **(D)** CD38 expression on the leukemic blasts from patient #3 and patient #5 treated with IRX195183.

One patient (Patient 5) achieved a morphological complete remission with incomplete hematologic recovery. He had AML secondary to MDS and had not responded to seven cycles of azacitidine 75 mg/m2, days 1–7. At baseline, he was pancytopenic, had 21% circulating blasts, and was red blood cell transfusion-dependent. Within 2 weeks of initiation of the study drug, his absolute neutrophil, and platelet counts had normalized. He also developed a leukocytosis with neutrophilic predominance, so he was treated with hydroxyurea. A bone marrow biopsy at 2 weeks demonstrated 15% blasts by immunohistochemistry and 33% by flow cytometry. At 1 month, his bone marrow biopsy demonstrated a hypercellular marrow with normal maturation and no morphological evidence of leukemia. Flow cytometric analysis detected 0.13% residual leukemic blasts, though the blasts demonstrated significantly increased expression of CD38, suggestive of maturation (**Figure 3D**). Although he developed recurrent thrombocytopenia before completion of cycle 1, he achieved transfusion independence, which he maintained for 2 months while on study. The study drug was held for Grade 3 hypertriglyceridemia on cycle 3 day 1. On cycle 3 day 9, he developed progressive disease and went off trial.

Two patients with MDS or CMML had stable disease while on IRX195183. Patient 6 had CMML with a baseline bone marrow demonstrating 2% blasts. He was treated on study for 63 days but withdrew from the study due to recurrent Grade 3 hypertriglyceridemia. His final bone marrow assessment showed CMML with no evidence of increased blasts, consistent with stable disease. Patient 8 had MDS that was unresponsive to six cycles of azacitidine prior to enrollment in the IRX195183 study. He was treated on study for five cycles and maintained stable disease until finally progressing to AML.

Two patients with AML had stable disease on their 1 month bone marrow specimens. Patient 1 withdrew from the study due to recurrent line-associated bacteremia. Patient 3 stopped the study drug due to hypertriglyceridemia and lack of response; however, she had evidence of significantly increased CD38 expression on the leukemic blasts from the bone marrow aspirate obtained at the end of cycle 1, suggestive of leukemic blast maturation (**Figure 3D**). Four patients discontinued the study drug due to progressive disease. Two patients were treated for less than one cycle due to infection and could not be formally evaluated for response, but they were considered non-responders for the purposes of the efficacy analysis.

### Plasma Differentiation Assay

To investigate if patients receiving IRX195183 achieve biologically meaningful plasma concentrations of this drug, we designed a functional assay to determine to what extent plasma from these patients can trigger differentiation *in vitro*. Using NB4 cells as a read out, 10% plasma from patients treated with IRX195183 induced a 5.8 ± 0.2 fold upregulation of CD11b expression and 52% (± 16%) decreased clonogenic activity compared to the control (**Figures 3A–C**). Plasma from patients with APL treated with ATRA had similar effects on NB4 cells, while plasma from normal volunteers had no discernable differentiation potential. For these experiments, we used plasma from Patients 1, 2, 3, and 4 both in DL1 and receiving 50 mg daily of IRX195183 and equivalent of 25 mg/m2 and 28 mg/m2, respectively. For comparison, we used plasma from patients with low-intermediate risk APL who received 45 mg/m2 of ATRA as part of standard-of-care Arsenic Trioxide (ATO) plus ATRA. The characteristics of the ATRA-treated patients and the timing of plasma sampling is shown in **Table 3**. Of note, at the time of plasma collection, these patients had yet received treatment with arsenic trioxide. Consistent with our previous observations that mesenchymal stroma cells block ATRA induced differentiation of APL cells, plasma from patients treated with ATRA showed no pro-differentiation activity on NB4 cells in the presence of stroma. In contrast, plasma from patients treated with IRX195183 continue to show induction of CD11b expression and reduction of clonogenic activity of NB4 cells in the presence of bone marrow stroma (**Figures 3A–C**). Compared to the effects of plasma from patients treated with ATRA, IRX195183 induced a greater increase in CD11b expression (t-test p < 0.01, Wilcoxon p = 0.057) and reduction in clonogenic activity (t-test p = 0.02, Wilcoxon p = 0.57) on NB4 cells cultured on bone marrow stroma.

**Table 3:** Characteristics of ATRA-treated patients from whom plasma samples were obtained and the timing of plasma sampling relative to last ATRA dose.

ATRA-treated Patients	Sex	Age	Diagnosis	WBC at diagnosis	Treatment protocol	Cycle and Day at the Time of Plasma Sampling	Time of Plasma Sampling Relative to Last ATRA Dose
1	M	64	APL	0.76	ATO + ATRA	Cycle 1, Day 2	1-h post dose 3 of ATRA
2	F	69	APL	7.57	ATO + ATRA	Cycle 1, Day 2	2-h post dose 3 of ATRA
3	M	67	APL	5.89	ATO + ATRA	Cycle 2, Day 2	2-h post dose 3 of ATRA

ATO, Arsenic trioxide.

## Discussion

In this phase 1 study, IRX195183 had a manageable toxicity profile and led to one short-lived morphological CRi response. The most significant toxicity associated with the drug was hypertriglyceridemia. This adverse effect led three patients to withdraw from the study, though none of these patients suffered adverse clinical sequelae. The incidence of hypertriglyceridemia observed in this study is comparable to the 47% incidence reported in studies of single-agent ATRA for the treatment of APL and to the 64% incidence reported in studies of single agent tamibarotene in patients with relapsed/refractory AML (23, 24). Anticipation of this adverse effect and prophylactic use of lipid modifying agents may be effective in mitigating this complication in future studies. Elevated liver enzymes were rare and self-limited in this study, in contrast to the results of an unpublished phase 1 clinical trial conducted in patients with hepatocellular carcinoma. There were no other DLTs or treatment-related deaths on study. Notably, the maximally tolerated dose of the drug was not reached in this study due to limited supply.

Furthermore, two patients showed *in vivo* maturation of their leukemic blasts, providing evidence that IRX195183 is clinically active. One of these patients also experienced a CRi response with an improvement in blood counts and transfusion independence at 1 month. The second patient did not respond clinically.

Nevertheless, we demonstrated that IRX195183 administered at the lowest dose level achieves concentrations sufficient to differentiate leukemic blasts *in vitro*. In contrast to plasma from patients who received ATRA, the differentiating effect of plasma from patients on IRX195183 was unaffected by the presence of stroma. The implication of this finding is that IRX195183 may be more effective at differentiating leukemic blasts within the bone marrow, a site that otherwise serves as a sanctuary site for residual disease.

Our study was limited primarily by its early termination due to the inadequate supply of study drug. As a result, the study did not meet its target for enrollment in phase I, and it was unable to proceed onto the phase II expansion cohort. The availability of the drug for future investigative and clinical uses is uncertain, however our experience with IRX195183 may inform the design of future studies aiming to incorporate retinoid analogs into the treatment of non-APL AML. In addition, the plasma differentiation potential assay described here captures the significant impact that bone marrow stroma has on retinoids pharmacodynamics and may serve as a platform for interrogating the biological activity of other novel differentiating agents.

Our group has recently demonstrated the mitigating effect of bone marrow stroma on the differentiating capacity of retinoids, an effect that is mediated by CYP26 expression. IRX195183 is resistant to metabolism by CYP26 and is selective for RARα and therefore does not trigger upregulation of CYP expression in the liver and bone marrow stroma (17, 25). The result is better maintenance of the systemic C_max_ and AUC than ATRA (see protocol for NCT02749708 on Clinicaltrials.gov). These properties may translate into higher concentrations within the bone marrow microenvironment, resulting in more effective differentiation of residual leukemic blasts within this compartment (26, 27). Tamibarotene is another novel retinoid-agonist with increased specificity and potency for RARα (28). Its plasma concentrations do not decline overtime, as is seen with ATRA, and it has a lower affinity for cellular retinoic acid binding protein, which is expressed as a resistance mechanism in ATRA-resistant APL (29). Tamibarotene has shown efficacy in relapsed/refractory APL and better outcomes in the maintenance setting in comparison to ATRA (24, 30, 31). The increased selectivity demonstrated by both IRX195183 and tamibarotene may reduce off target activation of other retinoic acid receptors, reducing the burden of adverse effects associated with retinoid therapy.

Our findings suggest that IRX195183 is a safe and potentially effective agent in the treatment of retinoid-sensitive AMLs. Further studies of the efficacy of IRX195183 and other retinoic acid agonists either as single agents or in combination with other therapies in the treatment of biologically defined non-APL AML are warranted.

## Author’s Note

RC passed away during preparation of the manuscript. Final version was approved on his behalf by MS, CEO of IO therapeutics.

## Data Availability Statement

The raw data supporting the conclusions of this article will be made available by the authors, without undue reservation.

## Ethics Statement

The studies involving human participants were reviewed and approved by the Johns Hopkins Institutional Review Board. The patients/participants provided their written informed consent to participate in this study. Written informed consent was obtained from the individual(s) for the publication of any potentially identifiable images or data included in this article.

## Author Contributions

AA was involved in data collection, analysis, interpretation, and writing. KN was involved in study design, provision of study patients, data collection, analysis, interpretation, and writing. DH, LP, BP, and AD performed the correlative studies. RC and MS were involved in study design. RV was involved in data analysis and interpretation. RJ was involved in writing. BS was involved in study design, provision of study patients, data analysis, interpretation, and writing. GG was involved in study design, data analysis, interpretation, and writing. All authors contributed to the article and approved the submitted version.

## Funding

This project was supported by the National Heart, Lung, and Blood Institute (grants K08-HL127269, R03 HL145226, T32-HL007525) (GG, LP), the National Cancer Institute (grants P01-CA225618, P30-CA00793, 5T32-CA009071) (BS, RJ, GG, AA), The Leukemia and Lymphoma Society (LLS TRP 14086277) (RJ), and The Augustine Fellowship (KN). IRX195183 was provided by Io Therapeutics, Inc.

## Conflict of Interest

MS, RC, RJ, and GG are authors on a patent application for the use of IRX195183.

The remaining authors declare that the research was conducted in the absence of any commercial or financial relationships that could be construed as a potential conflict of interest.

## Footnotes

1 https://clinicaltrials.gov/ProvidedDocs/08/NCT02749708/Prot_SAP_000.pdf [Accessed February 21, 2020]
